# Supplementary material for: Delivery Strategies of siRNA Therapeutics for Hair Loss Therapy
Source: Int J Mol Sci. 2024 Jul 11;25(14):7612. doi: 10.3390/ijms25147612 (PMC11277092; doi:10.3390/ijms25147612)
Supplement: Supplementary file 1 [file ijms-25-07612-s001.zip › ijms-3015952-supplementary.pdf]

International Journal of *Molecular Sciences*

MDPI

**Supplementary Table S1.** Delivery approaches based on key interventions in clinical trials for hair loss.

| Category | Key intervention | Dose/ Delivery system | Administration route | Condition | NCT number |
|---|---|---|---|---|---|
| Small molecules, corticosteroids and others | Finasteride | 1 mg | - | Androgenetic alopecia (female) | NCT01052870 |
| | Dutasteride | 0.02, 0.1 and 0.5 mg | Oral | Androgenetic alopecia | NCT01231607 |
| | | 0.5 mg | Oral | Male pattern hair loss or androgenetic alopecia | NCT02014584 |
| | Minoxidil | 5%, q.d. (foam)<br>2%, b.i.d. (solution) | Topical | Female pattern hair loss | NCT01145625 |
| | | 5% (90% ethanol and 5% propylene glycol) | Topical | Female pattern hair loss | NCT04090801 |
| | | 5% (foam) | Topical | Female pattern hair loss | NCT01226459 |
| | | 5% (noisome, spray) | Topical | Alopecia areata | NCT05587257 |
| | Methylprednisolone (sodium succinate) | 15 mg/kg (200 mL fresh orange juice) | Oral | Alopecia totalis<br>Alopecia universalis<br>Ophiasic alopecia | NCT01167946 |
| | Triamcinolone acetonide | 5 mg/mL | Intralesional | Alopecia areata | NCT03535233 |
| | Hydroxychloroquine | - | - | Alopecia areata | NCT00176982 |
| | ALRV5XR | 1 each (capsule)<br>3-7 mL (shampoo) | Oral<br>Topical | Androgenetic alopecia<br>Telogen effluvium<br>Hair thinning<br>Hair loss/baldness | NCT04450602 |
| | | 1 each (capsule)<br>3-7 mL (shampoo)<br>1 mL (serum) | Oral<br>Topical<br>Topical | Androgenetic alopecia<br>Telogen effluvium<br>Hair thinning<br>Hair loss/baldness | NCT04450589 |
| | Zinc supplement with minoxidil solution (5%) | 15 mg chelate zinc supplement: additional to 5% minoxidil (solution) | Oral | Female pattern hair loss | NCT01662089 |
| Jak inhibitors and antibodies | Ifidancitinib (ATI-50002) | 0.12 and 0.46% (solution) | Topical | Alopecia areata | NCT03354637 |
| | Baricitinib (LY3009104) | 2 and 4 mg (tablet) | Oral | Alopecia areata | NCT03899259 |
| | | 2 and 4 mg (tablet) | Oral | Severe or very severe alopecia areata | NCT03570749 |
| | | High and low (tablet) | Oral | Alopecia areata<br>Alopecia<br>Hypotrichosis<br>Hair diseases<br>Skin diseases | NCT05723198 |
| | Deuruxolitinib (CTP-543) | 8 and 12 mg (tablet) | Oral | Alopecia areata | NCT04797650 |
| | Jaktinib | 50 and 75 mg (tablet) | Oral | Alopecia areata | NCT05051761 |
| | | 50, 150 and 200 mg (tablet) | Oral | Alopecia areata | NCT04034134 |
| | | 0.5, 1.5 and 2.5% (cream) | Topical | Alopecia areata | NCT04445363 |
| | Ritlecitinib (PF-06651600) | 200 mg (q.d., 8 weeks) and then 100 mg (q.d., 40 weeks) (tablet) | Oral | Cicatricial alopecia | NCT05549934 |

| Category | Key intervention | Dose/ Delivery system | Administration route | Condition | NCT number |
|---|---|---|---|---|---|
| | Ruxolitinib | 20 mg (tablet) | Oral | Alopecia areata | NCT01950780 |
| | | 0.6% (cream) | Topical | Alopecia areata | NCT02553330 |
| | | Initial dose and maintenance dose | Oral | Autoimmune polyendocrinopathy candidiasis ectodermal dystrophy (APECED) Alopecia areata | NCT05398809 |
| | Tofacitinib | 5 – 10 mg | Oral | Alopecia areata | NCT02299297 |
| Cell therapy or cell therapy-related products | Human autologous hair follicle cells | - | Injection to scalp | Androgenetic alopecia | NCT01286649 |
| | Autologous cultured dermal and epidermal cells with 5% minoxidil | - | Injection to scalp | Androgenetic alopecia Male pattern baldness Female pattern baldness | NCT01451125 |
| | Adipose derived stem cell suspension plus platelet rich plasma | - | Follicular unit extraction | Androgenetic alopecia | NCT03388840 |
| | Hair stimulating complex | 0.1 mL (30-gauge needle) | Intradermal injection to scalp | Androgenetic alopecia | NCT01501617 |
| | Adipose-derived stromal vascular fraction (GID SVF-2) | - | Intradermal injection to scalp | Androgenetic alopecia | NCT02626780 |
| | Conditioned media of umbilical cord blood-derived stem cells (NGF-574H) | Hair serum with 5% conditioned media of umbilical cord blood-derived stem cells | Directly used by subjects themselves at home | Androgenetic alopecia | NCT03676400 |
| | Biocellular-cellular regenerative mixture | - | Intravenous infusion | Alopecia areata Scarring alopecia | NCT03078686 |
| | Autologous fat graft enriched with adipose-derived regenerative cells (ADRCs) | Purified adipose + 500,000 or + 1,000,000 ADRCs | Subcutaneous injection in scalp | Androgenetic alopecia | NCT02503852 |
| | Platelet-rich plasma | 6 mL, activated with or without pulsed electrical fields | Subcutaneous injection | Androgenetic alopecia | NCT05348343 |
| | | - | Injection to half head | Androgenetic alopecia | NCT02074943 |
| | | - | Injection to scalp | Androgenetic alopecia | NCT03376581 |
| Medical devices | Theradome LH80 pro for photobiomodulation therapy combined with scalp cooling | A wearable laser helmet device, thrice weekly | Light therapy to scalp | Chemotherapy-induced alopecia | NCT05177289 |
| | REVIAN 101 | A cap for portable use with rechargeable battery and adapter with active LEDs, a daily 10-minute treatment over the course of 26-weeks | Modulated light therapy to scalp | Androgenetic alopecia | NCT04019795 |
| | Derma pen | Microneedling combined with methotrexate (25 mg/mL) at 0.02 mL/cm2, a maximum | Topical | Alopecia areata | NCT05485571 |

| Category | Key intervention | Dose/ Delivery system | Administration route | Condition | NCT number |
|---|---|---|---|---|---|
| | | of 0.1-0.2 mL (2.5-5 mg) | | | |
| | | Microneedling after 5% minoxidil application | Topical | Alopecia areata | NCT05587257 |
| | Dermojet | A needless syringe, 0.1 mL of Depo-Medrol (methylprednisolone acetate) 40 mg/2 mL | Injection to scalp | Alopecia areata | NCT01017510 |
| | Dimethyl ether and propane (DMEP) kit | Superficial cryotherapy using DMEP at -57℃ | Topical | Alopecia areata | NCT04680234 |
| | MTS-01 | 7% (gel in 100-mL tube) | Topical | Radiotherapy-induced alopecia | NCT00713154 |
| | Paxman cooling machine | - | Topical | Chemotherapy-induced alopecia | NCT01008774 |
| | UV | Narrow band UVB (311 nm) phototherapy | Topical | Alopecia areata | NCT03847441 |
| | | UVB excimer light, twice weekly | Topical | Alopecia areata | NCT01802177 |
| | Venus glow™ | Venus Glow hydradermabrasion device (cleansing and micromassaging) | Hydradermabrasion on the scalp | Androgenetic alopecia | NCT05426629 |
| | HairDx | Sample collection kit for DNA | Saliva collection | Hair loss<br>Hair loss/baldness<br>Female pattern baldness<br>Androgenetic alopecia | NCT04379583 |
| Formulations or delivery systems | Aldara (imiquimod) | 5% (cream) | Topical | Alopecia areata | NCT00177021 |
| | Crisaborole | Ointment | Topical | Alopecia areata | NCT04299503 |
| | Diphenylcyclopropenone (DPCP) | Ointment | Topical | Alopecia areata | NCT03651752 |
| | LEO 124249 | Ointment | Topical (eyebrow) | Alopecia areata | NCT03325296 |
| | CU-40101 | Liniment | Topical | Androgenetic alopecia | NCT05380427 |
| | CU-40102 | 0.25% (2.275mg/mL) (spray) | Topical | Androgenetic alopecia | NCT05135468 |
| | XN-001 | Nitric oxide gel [14.6 mM sodium nitrite in distilled water with HEC[1] (m.w. 50,000-1,250,000);<br>14.6 mM maleic acid and 14.6 mM ascorbic acid in distilled water with HEC] | Topical | Androgenetic alopecia | NCT01347957 |
| | Targretin (bexarotene) | 1% (gel) | Topical | Alopecia areata | NCT00063076 |
| | Latanoprost | 0.005% (solution) | Topical | Alopecia areata | NCT02350023 |
| | | Ophthalmic solution | Topical (eyelash) | Alopecia areata | NCT00187577 |

| Category | Key intervention | Dose/ Delivery system | Administration route | Condition | NCT number |
|---|---|---|---|---|---|
| | Autologous platelet-rich fibrin matrix (PRFM) | 0.1 mL (4-8 mL, isolated from 9-18 mL of peripheral blood) | Intradermal injection to scalp | Alopecia | NCT01590238 |
| | Sodium valproate | Nanospanlastic dispersion | Topical | Alopecia areata | NCT05017454 |
| | Nanofat | Autologous nanofat grafting | Injection to scalp | Androgenetic alopecia | NCT03506503 |
| | Exosomes | 100e10 particle (exosomes isolated from human amniotic mesenchymal stem cells) | Injection | Alopecia<br>Hair loss (prevention) | NCT05658094 |

[1] Hydroxyethylcellulose
